# Editorial: Advances in Cardiac Pacing and Neural Control Strategies: Basic, Translational and Clinical Research

**DOI:** 10.3389/fphys.2022.866991

**Published:** 2022-02-28

**Authors:** Kanchan Kulkarni, Siamak Salavatian, Stavros Stavrakis

**Affiliations:** ^1^University of Bordeaux, INSERM, Centre de Recherche Cardio-Thoracique de Bordeaux, U1045, Bordeaux, France; ^2^IHU Liryc, Electrophysiology and Heart Modeling Institute, Fondation Bordeaux Université, Bordeaux, France; ^3^Department of Anesthesiology and Perioperative Medicine, University of Pittsburgh, Pittsburgh, PA, United States; ^4^Division of Cardiology, Department of Medicine, University of Pittsburgh, Pittsburgh, PA, United States; ^5^Heart Rhythm Institute, University of Oklahoma Health Sciences Center, Oklahoma City, OK, United States

**Keywords:** cardiac pacing, autonomic nervous system, neuromodulation, arrhythmias, alternans

Cardiovascular diseases are the major cause of mortality worldwide with over 17 million deaths occurring due to sudden cardiac death (SCD) annually. SCD instigated by cardiac arrhythmias accounts for over 300,000 deaths annually in the US alone (Srinivasan and Schilling, 2018). As aptly summarized by Patel et al., the prevalence of atrial tachyarrhythmias and atrial fibrillation (AF) in particular, has been increasing in the general US population with an estimated 59.7 million cases of AF or atrial flutter reported in 2019 (Lippi et al., 2021). This 33% increase in the prevalence of AF in the last 20 years, is predicted to increase >60% by 2050 (Lippi et al., 2021). About 1.5–5% of the general population is currently afflicted by arrhythmias (Desai and Hajouli, 2021), either atrial or ventricular, with ~90,000 new cases of supraventricular tachycardia being diagnosed annually.

Cardiac pacemakers have been around for over 60 years, and we have seen tremendous advances in anti-arrhythmic pacing techniques since then. The past couple of decades have also shone a light on the utility of neuromodulation techniques for the treatment of cardiovascular diseases. Autonomic modulation using vagal nerve stimulation (cervical or auricular), renal denervation or spinal cord stimulation has been shown to reduce infarct size, modulate cardiac alternans, demonstrate anti-arrhythmic and anti-inflammatory effects (Stavrakis et al., 2020). Despite these promising efforts, cardiac arrhythmias remain a significant burden on healthcare and account for about 20% of all deaths annually.

This Research Topic was initiated to provide a forum for investigators working on novel treatment strategies for cardiovascular diseases to report the current advances in cardiac pacing techniques. In addition, it provided a platform to demonstrate recent techniques in cardio-neurology and provide insights into approaches for controlling abnormal cardiac rhythms using autonomic modulation.

## Cardiac Pacing Techniques for Arrhythmia Detection and Prevention

In their review article, Patel et al. provide an overview of clinical arrhythmia management methods. They compare traditional treatment modalities with promising potential pacing techniques for predicting cardiac arrhythmias and effectively suppressing abnormal rhythms. They provide insights on novel non-linear dynamical approaches for arrhythmia prevention, utility of artificial intelligence (AI) based techniques for predicting cardiac abnormalities, and also offer a perspective on bridging the gap between basic and clinical science for the successful translation of promising anti-arrhythmic pacing strategies. Their review provides a detailed overview of traditional clinical methods used for arrhythmia diagnosis, such as Holter monitoring, cardiac mapping, tilt table tests, pharmacological interventions, and implantable devices like pacemakers and cardioverter defibrillators. Additionally, they provide an overview of promising preclinical and clinical studies investigating novel non-linear dynamical pacing modalities for arrhythmia management.

## Neuromodulation Therapies for Cardiac Diseases

Ma et al. present results from an interesting study investigating the effect of acute exposure to a hypoxic environment on the cardiopulmonary system. The autonomic nervous system plays a vital role in the acclimatization of the heart to external or internal stimuli. Their study investigated the longitudinal changes and recovery in heart rate variability (HRV) in young healthy adults on exposure to a simulated plateau environment. The participants were subjected to normoxic (~101 kPa) and hypoxic (4,000 m above sea level, ~62 kPa) environments, and the effect of pressure change on HRV was investigated. They demonstrated that participants in hypobaric hypoxia had significantly lower HRV time-domain metrics, substantially higher LF/HF ratio, significantly lower Poincaré plot parameters and significantly reduced refined composite multi-scale entropy curves. Their study shows that exposure to hypobaric hypoxic environment can result in increased heart rates and sympathetic activation.

In another novel application of vagus nerve stimulation, Kulkarni et al. demonstrated that non-invasive, low level tragus stimulation (LLTS), modulates microvolt T-wave alternans in patients with ischemic cardiomyopathy and heart failure. Cardiac alternans has been shown to be a marker of impending arrhythmias and heightened levels of T-wave alternans have been associated with an increased predisposition to ventricular tachyarrhythmias. Prior preclinical studies have demonstrated that inhibition of T-wave alternans could prevent the onset of fatal tachyarrhythmias. In this present study, acute LLTS was performed on 26 patients with ischemic cardiomyopathy (left ventricular ejection fraction <35%) and heart failure. LLTS was performed at 5 and 20 Hz for 15 min, in active patients, both during sinus rhythm and with concomitant atrial pacing at 100 bpm. Their results demonstrate that right atrial pacing at 100 bpm leads to significantly heightened T-wave alternans burden compared to sinus rhythm, with or without LLTS. In addition, acute LLTS at both 5 and 20 Hz was shown to increase T-wave alternans burden. This study highlights the potential of acute LLTS in modulating alternans burden and forms the basis for evaluating the chronic effect of LLTS on T-wave alternans and ventricular tachyarrhythmia burden in this patient population.

Sympathetic overdrive has been shown to be associated with ventricular tachyarrhythmias and SCD. In an exciting new study, Kuwabara et al. investigated the utility of dorsal root ganglion stimulation (DRGS) in reducing cardiac sympathoexcitation and in turn ventricular arrhythmogenicity. They tested the induction of cardiac sympathoexcitation in swine using ventricular extrastimulation, before and after DRGS. Their data demonstrate that DRGS at both 20 Hz and 1 kHz, decreases pacing-induced activation recovery intervals shortening, reduces arrhythmogenicity by flattening the slope of the electrical restitution curve, and also decreases the Tpeak-Tend/QTc ratio. This study highlights the anti-arrhythmic effects of thoracic DRGS at both low-frequency and high-frequency, demonstrating its potential for countering cardiac excitability during sympathetic overdrive.

In summary, the collection of articles in this dedicated Research Topic, provides a comprehensive assessment of the contemporary treatment modalities for cardiac arrhythmia prediction, prevention and control. Moreover, these papers present novel promising neuromodulation approaches for treatment of cardiovascular diseases that have the potential to push traditional boundaries and radically improve cardiac patient care. Technological advancements in signal processing, the advent of AI-based diagnostic approaches and the exponential rise in big data engineering have ushered in a new era of medical science. The novel dynamical approaches for rhythm management being investigated, point to an extremely promising future for cardiac pacing therapies. In conjunction, real-time remote patient monitoring and telehealth have enabled access to healthcare for patients in underserved and isolated rural areas while simultaneously expanding accessibility for urban patients as well (Kulkarni et al., 2021). A synergistic endeavor between engineers, scientists and physicians can enable the implementation of an AI-based telehealth framework with continuous ambulatory monitoring, prediction of impending cardiovascular events and prompt application of preventive electrical interventions. The futuristic realm where AI, engineering and medicine walk hand in hand, promises not only better quality, but longer and healthier lives.

## Author Contributions

All authors listed have made a substantial, direct, and intellectual contribution to the work and approved it for publication.

## Conflict of Interest

The authors declare that the research was conducted in the absence of any commercial or financial relationships that could be construed as a potential conflict of interest.

## Publisher's Note

All claims expressed in this article are solely those of the authors and do not necessarily represent those of their affiliated organizations, or those of the publisher, the editors and the reviewers. Any product that may be evaluated in this article, or claim that may be made by its manufacturer, is not guaranteed or endorsed by the publisher.
